# Supplementary material for: HIVprotI: an integrated web based platform for prediction and design of HIV proteins inhibitors
Source: J Cheminform. 2018 Mar 9;10:12. doi: 10.1186/s13321-018-0266-y (PMC5845081; doi:10.1186/s13321-018-0266-y)
Supplement: Supplementary file 2 — Additional file 2. Source code of HIVProtI web server. [file 13321_2018_266_MOESM2_ESM.zip › HIVprotI_Source-code/hivprotI/weka-3-6-6/doc/weka/core/tokenizers/Tokenizer.html]

Tokenizer


---


|  |  |  |
| --- | --- | --- |
| **Overview** \| **Package** \| **Class** \| **Tree** \| **Deprecated** \| **Index** \| **Help** \| **Weka's home** | |  |
| **PREV CLASS**   **NEXT CLASS** | **FRAMES**    **NO FRAMES**     **All Classes** |  |
| SUMMARY: NESTED \| FIELD \| CONSTR \| METHOD | DETAIL: FIELD \| CONSTR \| METHOD |  |


---


## weka.core.tokenizers Class Tokenizer

```
java.lang.Object
  weka.core.tokenizers.Tokenizer
```

**All Implemented Interfaces:**: java.io.Serializable, java.util.Enumeration, OptionHandler, RevisionHandler

**Direct Known Subclasses:**: AlphabeticTokenizer, CharacterDelimitedTokenizer

---

``` public abstract class Tokenizer extends java.lang.Object implements java.util.Enumeration, OptionHandler, java.io.Serializable, RevisionHandler ```

A superclass for all tokenizer algorithms.

**Version:**
:   $Revision: 1.3 $

**Author:**
:   FracPete (fracpete at waikato dot ac dot nz)

**See Also:**: Serialized Form

---

**Constructor Summary**

`Tokenizer()`


| **Method Summary** | |
| --- | --- |
| `java.lang.String[]` | `getOptions()`             Gets the current option settings for the OptionHandler. |
| `abstract  java.lang.String` | `globalInfo()`             Returns a string describing the stemmer |
| `abstract  boolean` | `hasMoreElements()`             Tests if this enumeration contains more elements. |
| `java.util.Enumeration` | `listOptions()`             Returns an enumeration of all the available options.. |
| `abstract  java.lang.Object` | `nextElement()`             Returns the next element of this enumeration if this enumeration object has at least one more element to provide. |
| `static void` | `runTokenizer(Tokenizer tokenizer, java.lang.String[] options)`             initializes the given tokenizer with the given options and runs the tokenizer over all the remaining strings in the options array. |
| `void` | `setOptions(java.lang.String[] options)`             Sets the OptionHandler's options using the given list. |
| `abstract  void` | `tokenize(java.lang.String s)`             Sets the string to tokenize. |
| `static java.lang.String[]` | `tokenize(Tokenizer tokenizer, java.lang.String[] options)`             initializes the given tokenizer with the given options and runs the tokenizer over all the remaining strings in the options array. |

**Methods inherited from class java.lang.Object**

`equals, getClass, hashCode, notify, notifyAll, toString, wait, wait, wait`

**Methods inherited from interface weka.core.RevisionHandler**

`getRevision`

**Constructor Detail**

### Tokenizer

```
public Tokenizer()
```


**Method Detail**

### globalInfo

```
public abstract java.lang.String globalInfo()
```

:   Returns a string describing the stemmer

    :   **Returns:**: a description suitable for displaying in the explorer/experimenter gui

---


### listOptions

```
public java.util.Enumeration listOptions()
```

:   Returns an enumeration of all the available options..

    :   **Specified by:**: `listOptions` in interface `OptionHandler`
    :   **Returns:**: an enumeration of all available options.

---


### getOptions

```
public java.lang.String[] getOptions()
```

:   Gets the current option settings for the OptionHandler.

    :   **Specified by:**: `getOptions` in interface `OptionHandler`
    :   **Returns:**: the list of current option settings as an array of strings

---


### setOptions

```
public void setOptions(java.lang.String[] options)
                throws java.lang.Exception
```

:   Sets the OptionHandler's options using the given list. All options
    will be set (or reset) during this call (i.e. incremental setting
    of options is not possible).

    :   **Specified by:**: `setOptions` in interface `OptionHandler`
    :   **Parameters:**: `options` - the list of options as an array of strings **Throws:**: `java.lang.Exception` - if an option is not supported

---


### hasMoreElements

```
public abstract boolean hasMoreElements()
```

:   Tests if this enumeration contains more elements.

    :   **Specified by:**: `hasMoreElements` in interface `java.util.Enumeration`
    :   **Returns:**: true if and only if this enumeration object contains at least one more element to provide; false otherwise.

---


### nextElement

```
public abstract java.lang.Object nextElement()
```

:   Returns the next element of this enumeration if this enumeration object
    has at least one more element to provide.

    :   **Specified by:**: `nextElement` in interface `java.util.Enumeration`
    :   **Returns:**: the next element of this enumeration.

---


### tokenize

```
public abstract void tokenize(java.lang.String s)
```

:   Sets the string to tokenize. Tokenization happens immediately.

    :   **Parameters:**: `s` - the string to tokenize

---


### tokenize

```
public static java.lang.String[] tokenize(Tokenizer tokenizer,
                                          java.lang.String[] options)
                                   throws java.lang.Exception
```

:   initializes the given tokenizer with the given options and runs the
    tokenizer over all the remaining strings in the options array. If no
    strings remained in the option string then data is read from stdin, line
    by line.

    :   **Parameters:**: `tokenizer` - the tokenizer to use: `options` - the options for the tokenizer **Returns:**: the tokenized strings **Throws:**: `java.lang.Exception` - if setting of options or tokenization fails

---


### runTokenizer

```
public static void runTokenizer(Tokenizer tokenizer,
                                java.lang.String[] options)
```

:   initializes the given tokenizer with the given options and runs the
    tokenizer over all the remaining strings in the options array. The
    generated tokens are then printed to stdout. If no strings remained
    in the option string then data is read from stdin, line by line.

    :   **Parameters:**: `tokenizer` - the tokenizer to use: `options` - the options for the tokenizer


---


|  |  |  |
| --- | --- | --- |
| **Overview** \| **Package** \| **Class** \| **Tree** \| **Deprecated** \| **Index** \| **Help** \| **Weka's home** | |  |
| **PREV CLASS**   **NEXT CLASS** | **FRAMES**    **NO FRAMES**     **All Classes** |  |
| SUMMARY: NESTED \| FIELD \| CONSTR \| METHOD | DETAIL: FIELD \| CONSTR \| METHOD |  |


---
